# Real-Time Phase-Sensitive OTDR Based on Data Matrix Matching Method

**DOI:** 10.3390/s18061883

**Published:** 2018-06-08

**Authors:** Xin Liu, Yu Wang, Ruidong Wu, Dong Wang, Qing Bai, Baoquan Jin

**Affiliations:** 1 Key Laboratory of Advanced Transducers and Intelligent Control Systems, Taiyuan University of Technology, Ministry of Education and Shanxi Province, Taiyuan 030024, China; liuxin0127@link.tyut.edu.cn (X.L.); wangyu@tyut.edu.cn (Y.W.); wuruidong0881@link.tyut.edu.cn (R.W.); wangdong@tyut.edu.cn (D.W.); baiqing0122@link.tyut.edu.cn (Q.B.); 2 State Key Laboratory of Coal and CBM Co-mining, Jincheng 048012, China

**Keywords:** data matrix matching method, real-time vibration location, type identification, Φ-OTDR

## Abstract

Phase-sensitive optical time-domain reflectometry (Φ-OTDR) is an effective technique to accomplish fully distributed vibration measurement along the entire fiber link. In this paper, a novel data matrix matching method is proposed and successfully employed in the Φ-OTDR system for real-time vibration detection and type identification. By using the novel method, the quantized response time is presented and improved to millisecond level for the first time. Meanwhile, the data can be extracted completely without packet loss, thus allowing vibration type identification to be obtained while maintaining the system simplicity. The experimental results demonstrate that the vibration signals can be detected and located with an average response time of 50.1 ms, under a data transmission speed which can go up to 77.824 Mbps. Moreover, different vibration types such as sine waves and square waves which are applied to the sensing fiber through a piezoelectric ceramic (PZT) cylinder can also be successfully identified. This method provides an efficient solution for real-time vibration location and type identification, thus exhibiting considerable application potential in many practical situations.

## 1. Introduction

Distributed fiber-optic vibration sensors based on Φ-OTDR have received substantial research attention in recent years owing to their distinct advantages of high sensitivity, accurate positioning, good spatial resolution, and simultaneous multi-point monitoring capability [1,2,3]. Therefore, they exhibit huge potential in various applications such as intruder detection [4], structural health monitoring [5] and oil/gas pipelines integrity threat prevention [6], etc. The operating principle of the Φ-OTDR technique is based on detection and location of the phase changes of the Rayleigh back-scattering (RBS) light. In an Φ-OTDR system, a highly coherent laser instead of the traditional broadband light source is adopted, hence, the received RBS signal is modulated in a jagged appearance owing to the coherent interaction of numerous Rayleigh scattering centers within the injected pulse duration [7]. When a certain part of the sensing fiber is subject to an external vibration, the refractive index and/or the length of the sensing fiber will change at that position, thus resulting in a localized phase change in the light wave. Therefore, the intensity of the interference light will change at the time corresponding to the vibration position [8].

For an Φ-OTDR system, the basic function is to locate the vibration position, which can be obtained by several methods such as the traditional difference value method [9], the edge detection method [10], and the wavelet packet transform based method [11], etc. Usually, the direct detection method is adopted, since it is simple and robust [12]. Moreover, rapid response to vibration events is of crucial importance in practical situations. In the previous literatures [13,14], real-time detection is mentioned, but not quantized. For the commercially available products, the responses time is approximately 2 s. Thus, there still remains a need for a faster and quantized response time for the Φ-OTDR system.

In addition, with the development of Φ-OTDR, the mere position detection can hardly satisfy practical needs. In some occasions, not only the vibration position, but also the vibration type, e.g., frequency and relative amplitude, is needed to comprehensively judge a vibration signal. In 2010, Bao et al. proposed a coherent detection method, where the signal-to-noise-ratio (SNR) was improved due to the coherent amplification effect through controlling the power of local oscillator [15]. Based on the coherent scheme, the vibration type can be obtained by analyzing the beat portion of coherent detection [16]. Another method to achieve vibration type is merging interferometers, such as the Mach-Zehnder interferometer (MZI) [17,18] and Michelson interferometer (MI) [19,20]. In these systems, the vibration position was located by the Φ-OTDR architecture, while the frequency and amplitude information was achieved by demodulating the interference signal. For the coherent detection method and the combining Φ-OTDR with interferometers method, more devices are required, which increases the complexity and cost. In comparison, the direct detection method is less complex, simpler, and more robust. In recent years, some investigations in regard to the vibration type are conducted utilizing the direct detection method. For instance, a graphics processing unit-based parallel computing method was proposed to perform space-frequency analysis [21]. Muanenda et al. achieved high-frequency vibration information with minimal post-processing by suitable design of the amplification and modulation of the pulses [22].

In this paper, a novel data matrix matching method is proposed and employed in the direct Φ-OTDR sensing system. Firstly, the original data stream is transformed into the matrix form through data synchronization. Subsequently, the data dimensionality reduction is conducted to improve the system response performance. Finally, the effective data is completely transmitted to the host computer for real-time vibration detection and type identification.

This article is structured as follows: the underlying principle of the proposed method is detailed in Section 2, followed by a description of the system implementation in Section 3. Experimental results are presented and discussed in Section 4. Finally, the conclusions are drawn in Section 5.

## 2. Theoretical Analysis

When the light pulse propagating in the sensing fiber, the RBS traces are generated. Assuming that {*P*_1_, *P*_2_, …, *P_N_*} is *N* probe pulses emitted from the laser within one second, and {*X*_1_, *X*_2_, …, *X_N_*} is the corresponding raw RBS traces, which can be expressed as:

(1) $$X_{raw}=\begin{array}{cccccc} {}[X_{1} & X_{2} & \cdots & \;X_{i}\; & \cdots & X_{N}] \end{array},\;i\in \left(1,N\right)$$

where *X_i_* = [*x_i_*_1_, *x_i_*_2_, …, *x_iM_*], which represents the *i*-th reflectogram caused by the *i*-th probe pulse. The amount of raw data generated by the probe pulse within one second used to monitor the vibration response of the entire fiber is massive. In this case, data synchronization of the reflectograms is of great importance in order to comprehensively judge the vibration information. Therefore, a data flag is added to each RBS trace, that is *X’_i_* = [*x_f_*, *x_i_*_1_, *x_i_*_2_, …, *x_iM_*]. Subsequently, the obtained signal *X* within one second can be expressed in a matrix form, that is:

(2) $$X=X_{raw}^{{}^{\prime }T}=\left[\begin{array}{c} X_{1}^{\prime }\\ X_{2}^{\prime }\\ \vdots \\ X_{i}^{\prime }\\ \vdots \\ X_{N}^{\prime } \end{array}\right]=\left[\begin{array}{ccccccccccc} x_{f} & x_{11} & x_{12} & \cdots & x_{1j} & \cdots & x_{1k} & \cdots & x_{1r} & \cdots & x_{1M}\\ x_{f} & x_{21} & x_{22} & \cdots & x_{2j} & \cdots & x_{2k} & \cdots & x_{2r} & \cdots & x_{2M}\\ \vdots & \vdots & \vdots & & \vdots & & \vdots & & \vdots & & \vdots \\ x_{f} & x_{i1} & x_{i2} & \ldots & x_{ij} & \cdots & x_{ik} & \cdots & x_{ir} & \cdots & x_{iM}\\ \vdots & \vdots & \vdots & & \vdots & & \vdots & & \vdots & & \vdots \\ x_{f} & x_{N1} & x_{N2} & \cdots & x_{Nj} & \cdots & x_{Nk} & \cdots & x_{Nr} & \cdots & x_{NM} \end{array}\right]$$

Thus, in Equation (2), the row vector is related to the sensing distance/time, while the column vector represents the repeated measurements under different light pulses. Supposing that *f_p_* is the pulse repetition frequency and *f_s_* is the sampling frequency, the number of rows *N* and columns *M* within one second are given by:

(3) $$\{\begin{array}{c} M=\frac{f_{s}}{f_{p}}\\ N=f_{p} \end{array}$$

Subsequently, the *k*-th column vector which corresponds to the sensing fiber end can be expressed as:

(4) $$k=\frac{2nf_{s}l_{0}}{c}$$

where *l*_0_ is sensing fiber length, *n* is the refractive index, and *c* is the light speed in vacuum. Thus, during one second time interval, the original signal matrix *X* includes two parts: *X_s_* and *X_v_*, which can be respectively extracted by introducing matrix *S* and *V*:

(5) $$\{\begin{array}{c} X_{s}=X\cdot S=\left[\begin{array}{ccccccc} x_{f} & x_{11} & x_{12} & \cdots & x_{1j} & \cdots & x_{1k}\\ x_{f} & x_{21} & x_{22} & \cdots & x_{2j} & \cdots & x_{2k}\\ \vdots & \vdots & & & & & \\ x_{f} & x_{i1} & x_{i2} & \cdots & x_{ij} & \cdots & x_{ik}\\ \vdots & \vdots & & & & & \\ x_{f} & x_{N1} & x_{N2} & \cdots & x_{Nj} & \cdots & x_{Nk} \end{array}\right]\\ X_{v}=X\cdot V=\left[\begin{array}{cccccc} x_{1(k+1)} & x_{1(k+2)} & \cdots & x_{1r} & \cdots & x_{1M}\\ x_{2}{}_{\left(k+1\right)} & x_{2(k+2)} & \cdots & x_{2r} & \cdots & x_{2M}\\ \vdots & \vdots & & \vdots & & \vdots \\ x_{i(k+1)} & x_{i(k+2)} & \cdots & x_{ir} & \cdots & x_{iM}\\ \vdots & \vdots & & \vdots & & \\ x_{N(k+1)} & x_{N(k+2)} & \cdots & x_{Nr} & \cdots & x_{NM} \end{array}\right] \end{array}$$

where *S* is an (*M* + 1) × (*k* + 1) matrix composed by a (*k* + 1) × (*k* + 1) diagonal matrix (*A*) and an (*M* − *k*) × (*k* + 1) null matrix (*B*). *V* is an (*M* + 1) × (*M* − *k*) matrix composed by a (*k* + 1) × (*M* − *k*) null matrix (*C*) and an (*M* − *k*) × (*M* − *k*) diagonal matrix (*D*), as shown in Equations (6) and (7):

(6) $$\{\begin{array}{c} S={\left[\begin{array}{c} A\\ B \end{array}\right]}_{\left(M+\;1)\;\times \;(k\;+\;1\right)}={\left[\begin{array}{cccc} 1 & 0 & \cdots & 0\\ 0 & 1 & \cdots & 0\\ \vdots & & & \\ 0 & 0 & \cdots & 1\\ 0 & 0 & \cdots & 0\\ 0 & 0 & \cdots & 0\\ \vdots & & & \\ 0 & 0 & \cdots & 0 \end{array}\right]}_{\left(M\;+\;1)\;\times \;(k\;+\;1\right)}\\ A={\left[\begin{array}{cccc} 1 & 0 & \cdots & 0\\ 0 & 1 & \cdots & 0\\ \vdots & & & \\ 0 & 0 & \cdots & 1 \end{array}\right]}_{\left(k\;+\;1)\;\times \;(k\;+\;1\right)};B={\left[\begin{array}{cccc} 0 & 0 & \cdots & 0\\ 0 & 0 & \cdots & 0\\ \vdots & & & \\ 0 & 0 & \cdots & 0 \end{array}\right]}_{\left(M\;-\;k)\;\times \;(k\;+\;1\right)} \end{array}$$

and:

(7) $$\{\begin{array}{c} V={\left[\begin{array}{c} C\\ D \end{array}\right]}_{{}_{\left(M\;+\;1)\;\times \;(M-k\right)}}={\left[\begin{array}{cccc} 0 & 0 & \cdots & 0\\ 0 & 0 & \cdots & 0\\ \vdots & & & \\ 0 & 0 & \cdots & 0\\ 1 & 0 & \cdots & 0\\ 0 & 1 & \cdots & 0\\ \vdots & & & \\ 0 & 0 & \cdots & 1 \end{array}\right]}_{\left(M+1)\;\times \;(M-k\right)}\\ C={\left[\begin{array}{cccc} 0 & 0 & \cdots & 0\\ 0 & 0 & \cdots & 0\\ \vdots & & & \\ 0 & 0 & \cdots & 0 \end{array}\right]}_{\left(k+1)\;\times \;(M-k\right)};D={\left[\begin{array}{cccc} 1 & 0 & \cdots & 0\\ 0 & 1 & \cdots & 0\\ \vdots & & & \\ 0 & 0 & \cdots & 1 \end{array}\right]}_{\left(M-k)\;\times \;(M-k\right)} \end{array}$$

Then, in order to improve the system real-time performance, although the raw trace set *X* is acquired, only the effective portion *X_s_* is transmitted for post-processing. The total data amount in bits of *X* and *X_s_* within one second is given in Equation (8):

(8) $$\{\begin{array}{c} V_{X}=(M+1)\times N\times 16\approx MN\times 16=16f_{s}\\ V_{s}=(k+1)\times N\times 16\approx kN\times 16=\frac{32nf_{s}f_{p}l_{0}}{c}=\eta \cdot V_{X} \end{array}$$

where *η* = 2*nf_p_l*_0_/*c* and thus is smaller than 1. Hence, the transmitted data amount is reduced by *η* times. In order to achieve complete data transmission without packet loss, the transmission speed should be no less than *V_s_* bps. As shown in Equation (8), *V_s_* is determined by the parameters of *n*, *f_s_*, *f_p_*, *l*_0_ and *c*. Among them, *n* and *c* are constants, thus, *V_s_* is actually determined by *f_p_*, *f_s_*, and *l*_0_. That is, if the transmission speed matches with the parameters of the data matrix, the effective data can be completely transmitted without packet loss. Practically, due to existence of the upper limit of transmission speed of the interface, the maximum sensing fiber length, and thus the value of *k* used in constructing the two matrices, is limited.

In addition, since the data is completely transmitted, *X_e_* contains the full effective information of the RBS signal *X*. Assuming that an external vibration with a frequency *f_v_* applied on a certain point of the fiber, taking the *j*-th column vector *x_j_* of the matrix:

(9) $$x_{j}=\left\{\begin{array}{c} x_{1j}\\ x_{2j}\\ \vdots \\ x_{ij}\\ \vdots \\ x_{Nj} \end{array}\right\}$$

Thus, the detected RBS signal will change at that position which corresponding to the *j*-th column vector. The sampling frequency is proportional to the number of RBS traces within one second, which reaches the upper limit (*f_p_*) in our system, such that:

(10) $$2f_{v}\leq f\leq f_{\max}=f_{p}$$

## 3. System Implementation

### 3.1. Hardware Design

**Figure 1:**
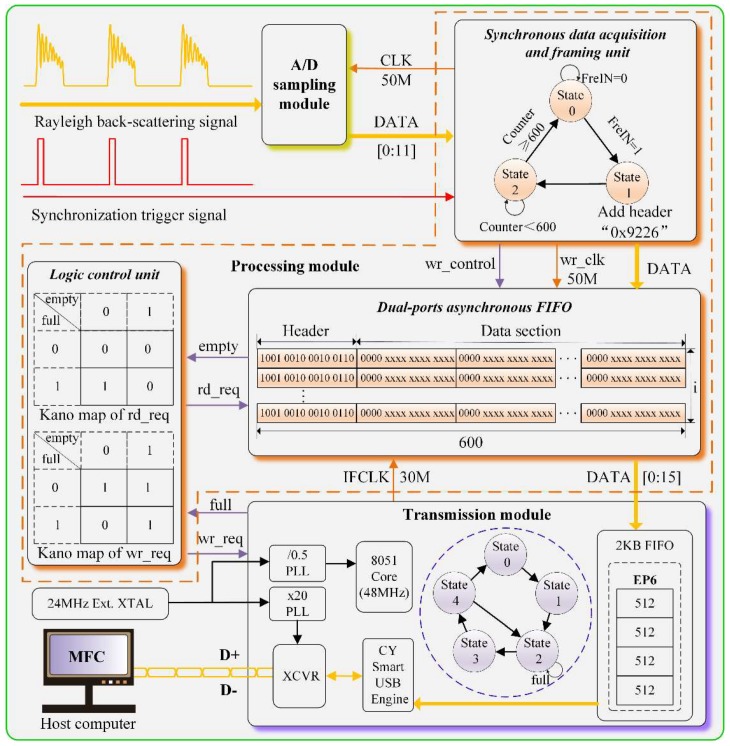
Overall block diagram of the hardware system.

In order to implement the proposed method, the hardware system is elaborately designed. The overall block diagram is presented in Figure 1, which mainly includes the A/D sampling module, the processing module and the transmission module, etc.

#### 3.1.1. A/D Sampling Module

In the proposed system, an AD9226 unit (Analog Devices, Inc., Norwood, MA, USA) with 12-bit accuracy is adopted as the analog to digital converter. In addition, “2 V Single-Ended Input” mode, of which the input range of AD9226 is 1~3 V, is chosen [23]. As shown in Figure 1, the input signal of A/D sampling module is the RBS light, which is detected by a Photo detector (PD). In our system, 2117FC (New Focus, Inc., San Jose, CA, USA) is utilized, of which the output voltage *V*_1_ can be expressed as [24]:

(11) $$V_{1}=(P_{+}-P_{-})\cdot R\cdot G$$

where *P*_+_ and *P*_−_ are the input optical powers of the right and left side in Watts. *R* is the response factor in V/mW, and *G* is the amplifier’s gain setting. In our experiment, the measured RBS light power is 44.7 nW, and the PD on the 3 × 10^3^ gain setting will have an output voltage approximately given by Equation (12):

(12) $$V_{1}=(44.7\;\mathrm{nW}-0)\times (1.07/1.5\;V/\mathrm{mW})\times (3\times 10^{3})=0.095\;V$$

**Figure 2:**
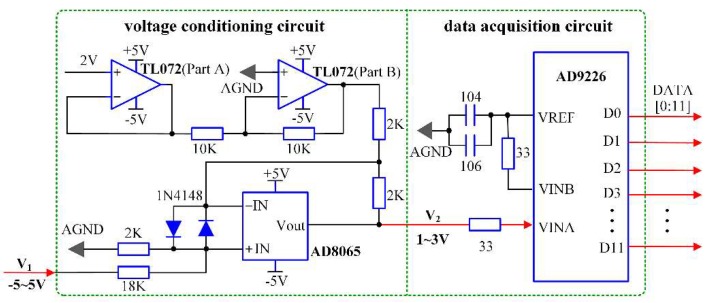
A/D sampling module.

Therefore, the calculated results reveal that the voltage *V*_1_ is not within the allowable input range of AD9226, thus, a voltage conditioning circuit is designed to satisfy the requirements of input voltage before the process of data acquisition. The A/D sampling module is illustrated in Figure 2.

As shown in Figure 2, both part A and part B of TL072 (Texas Instruments, Dallas, TX, USA) are operating at the voltage follower mode to produce the −2 V reference voltage for the AD8065 operational amplifier (Analog Devices, Inc. Norwood, MA, USA). Then, by analyzing the working mechanism of AD8065, the voltage conversion formula can be derived as follows:

(13) $$V_{2}=\frac{1}{5}V_{1}+2$$

As can be seen from Equation (13), *V*_2_ is 2.019 V when the input voltage *V*_1_ is 0.095 V, which can meet the input range of the AD9226. Thus, the initial voltage is adjusted to an appropriate range through the voltage conditioning circuit. Then, the signal is sent to AD9226, where the RBS light is sampled and converted to 12-bits digital signal.

#### 3.1.2. Processing Module

The processing module mainly consisted of the synchronous data acquisition and framing unit, the dual-ports asynchronous First-in First-out (FIFO) and the logic control unit, etc. As can be seen from Figure 1, the data from A/D sampling module is firstly sent to the synchronous data acquisition and framing unit, of which the operating principle is based on a state machine conversion. State 0 is the initial state, where the synchronous trigger signal has not arrived (FrqIN = 0). However, if the synchronous trigger signal is once captured (FrqIN = 1), the state machine will move to State 1, the ready state, where the data header “0x9226” is added to each frame. Afterwards, the state machine automatically turns to State 2 for high-speed data acquisition.

In our experiment, the sampling rate is 50 MS/s, corresponding to 2 m spatial distance. The total sensing fiber length is 1.2 km, hence, the effective sampling points is 600. If the sampling points “Counter” is smaller than 600, the state machine stays in the acquisition state, otherwise, it returns to the initial state. Therefore, only the effective portion of the RBS signal is selected and sent to the processing units, which greatly reduces the amount of data and alleviates the burden on both transmission and software.

Afterwards, the framing data packet is sent to the dual-ports asynchronous FIFO, of which the input clock is 50 MHz (wr_clk) and the output clock is 30 MHz (IFCLK), respectively. The frames are successively sent into the FIFO, that is, the *i*-th frame corresponds to *i*-th RBS trace. The data in FIFO is finally sent to the transmission module under the function of the logic control unit. For the dual-ports asynchronous FIFO, the input clock is 50 MHz, and the effective sampling points is 600, thus the sampling time is 12 μs. The output clock is 30 MHz, hence, the required output time of FIFO is 20 μs. In our design, the pulse repetition rate is 8 kHz, which ensures that the data can be completely transmitted within one pulse period.

The logic control unit is of crucial importance in the process of data transfer between the dual-ports asynchronous FIFO and the transmission module. In general, there are four control signals: “empty”, “full”, “rd_req” and “wr_req”. The Kano map of “rd_req” (active high) and “wr_req” (active low) are also shown in Figure 1. When the FIFO is not “empty” (active high) and transmission module is not “full” (active low), the data from FIFO can be read under the function of “rd_req” control signal and written into the transmission module under the function of the “wr_req” control signal. Therefore, only in the condition that FIFO is not “empty” (0) and transmission module is not “full” (1), can the data be transferred from the dual-ports asynchronous FIFO to the transmission module under the control of “rd_req” signal and “wr_req” signal, which ensures the integrity of the signal and effectively prevent data loss during the transmission process.

#### 3.1.3. Transmission Module

In the transmission module, a high-speed data transmission interface (HDIF) microcontroller CY7C68013A (Cypress, San Jose, CA, USA) is adopted. Generally, the HDIF can operate at two signaling speed modes: full speed and high speed. Considering the large data transmission requirement, high speed mode is selected. In addition, there are two working modes, namely general programmable interface (GPIF) mode and slave FIFO mode. In order to better cooperate with the processing module, the slave FIFO mode is employed in our design.

As can be seen from Figure 1, the working mechanism of transmission module is also based on a state machine conversion. State 0 is the idle state, which moves to State 1 when the data comes. State 1 is the Endpoint (EP) selecting state, and in our design, EP 6 mode with four 512 byte buffers is selected. Then the state machine moves to State 2, the full flag judging state. If the transmission module is not full, it moves to State 3, otherwise, it remains in State 2. State 3 is the data writing state, where the data is written into the corresponding FIFO under the control of the logic signal, and then the state machine moves to State 4, the jumping state. In this state, if there is still data to write, it moves to State 2, else case it returns to State 0. Later, the data is sent to the transceiver unit (XCVR) through the CY smart USB (universal serial bus) engine. Finally, two differential signals D+ and D− are sent to the host computer through XCVR. In addition, a 24 MHz external crystal oscillator provides work clock through different phase locked loops (PLL) for the enhanced 8051 core and XCVR, respectively. 

Therefore, by the collaboration of the A/D sampling module, the processing module and the transmission module, the effective data of the RBS signal is completely transmitted to the host computer.

### 3.2. Software Implementation

**Figure 3:**
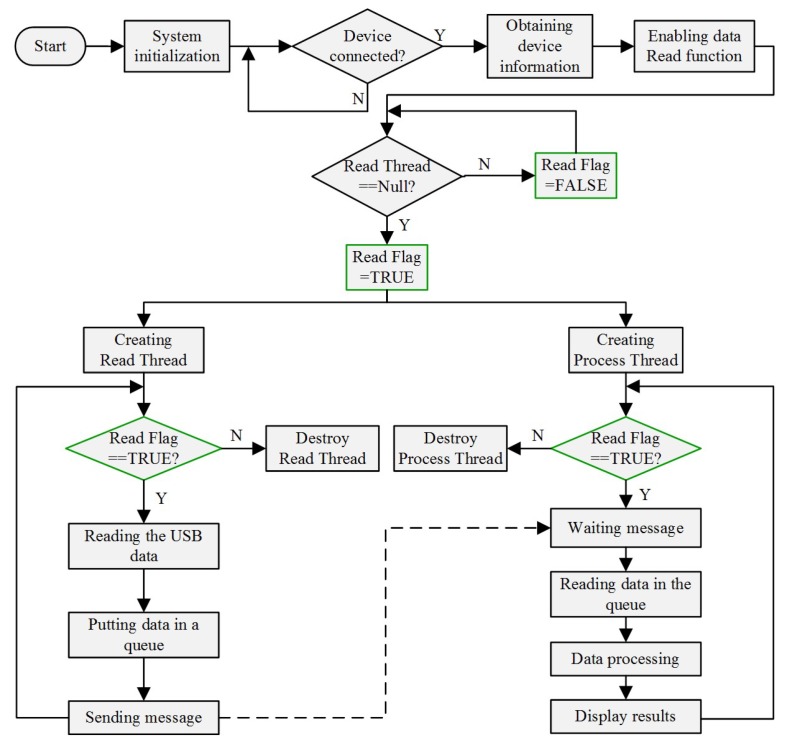
Software flow chart of real-time vibration detection based on MFC.

Due to the high execution efficiency and strong ability to establish a graphical interface, Microsoft Foundation Classes (MFC) has attracted wide attention and become one of the most popular tools for scientific researches in recent years. Therefore, MFC of Visual Studio 2013 is adopted as the coding tool in our system. In order to improve the real-time performance, the software is designed based on the technique of dual-thread mechanism, and real-time vibration detection is implemented in visual C++ under the Windows environment. The software flow chart of real-time vibration detection based on MFC is presented in Figure 3.

As depicted in Figure 3, the operating mechanism of the software is as follows: (1) System initialization, mainly including the initialization of the USB device, control signals, flag bits and Tee chart, etc. (2) Then, the software checks whether the USB device is connected or not. If it is connected, the device information such as vendor identification (VID) and product identification (PID) is obtained. Then, if the “Read” button is clicked, the data “Read” function is thereby enabled. Subsequently, the software checks if the “Read Thread” exists or not, in the case it doesn’t exist, two threads (the “Read Thread” and “Process Thread”) are created, respectively. (3) Afterwards, the software checks the status (“TRUE” or “FALSE”) of the “Read Flag”, if it is “TRUE”, the data is read and put in a queue, and finally a message is sent to the “Process Thread”. Once the “Process Thread” receives the message sent from the “Read Thread”, the data in the queue is read, processed and finally displayed on the MFC software interface. (4) If the “Read” button is cancelled, the order “Read Flag = FALSE” is then executed. Thus both the “Read Thread” and “Process Thread” are destroyed. In summary, based on the technique of the dual-thread mechanism, the system process efficiency is greatly promoted, which is propitious to achieve real-time vibration detection.

## 4. Experimental Results and Discussion

**Figure 4:**
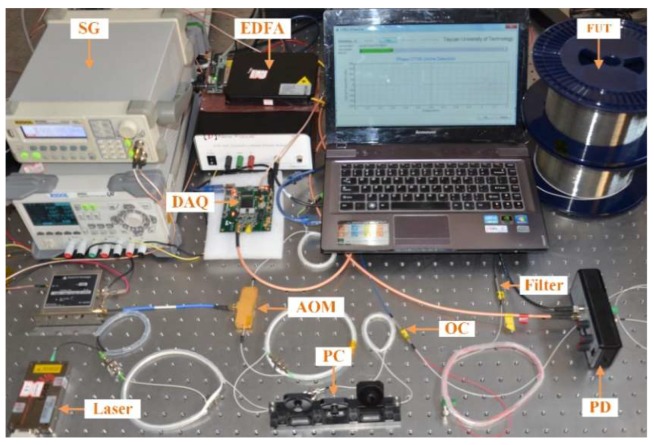
Experimental setup.

Preliminary experiments were performed in the laboratory to establish the feasibility of the proposed method. The experimental setup is demonstrated in Figure 4. A narrow line-width laser (≈3 kHz) with an output power of 20 mW is adopted as the light source. The state of polarization is scrambled using a polarization controller (PC) to adjust the polarization of the light. Then, the continuous wave is modulated into narrow pulses of 200 ns through an acoustic optical modulator (AOM), which is driven by a signal generator (SG). Afterwards, the modulated light pulses are amplified by an erbium-doped fiber amplifier (EDFA), and finally launched into the fiber under test (FUT) via an optical circulator (OC). Finally, the coherent RBS light is detected by the PD, processed by the data acquisition card (DAQ) and the results are displayed on the upper system.

### 4.1. Real-Time Performance Test

Firstly, a real-time performance test of the proposed system is conducted. In the experiment, the sensing fiber length is 1.2 km, and hence the light propagating time of a round trip in the sensing fiber *t*_1_ is 12 μs. In addition, the pulse repetition period *t*_2_ is 125 μs (corresponding to 8 kHz repetition rate). Then, the RBS light is detected and converted to the electrical signal by the PD. Afterwards, the electrical signal is sampled by A/D module with a sampling rate of 50 MS/s. Hence, the sampling time *t*_3_ is 0.02 μs. Eventually, the data is transmitted to the host computer.

**Figure 5:**
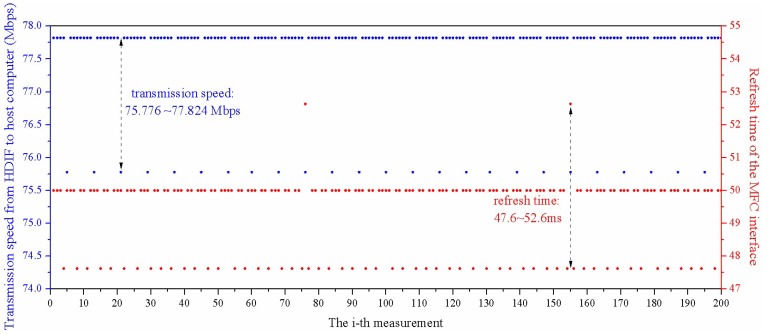
Experimental tests of the transmission speed and refresh time of the MFC interface.

Figure 5 shows the 200 tests of transmission speed. As can be seen from Figure 5, the transmission speed is within the range of 75.776 Mbps and 77.824 Mbps, hence, the transmission time *t*_4_ is approximately 125 μs (16 bits per point, 600 effective sampling points). Therefore, the experimental results agree quite well with the theoretical analysis, that is, the transmission speed matches with the parameters of the data matrix preset in the experiment, which ensures complete transmission of the effective data. Finally, the processed results are displayed on the MFC interface of the host computer.

Thus, based on the previous analysis, the response time of our system is at the level of microsecond theoretically. However, the practical response time is restricted by the refresh time of the MFC software interface, indeed. As shown in Figure 5, the refresh time of the MFC interface *t*_5_ is at millisecond level, which is far greater than the sum of *t*_1_, *t*_2_, *t*_3_ and *t*_4_. As shown in Figure 5, the refresh time is between the range of 47.6 ms and 52.6 ms. Therefore, the average response time of the our Φ-OTDR sensing system is 50.1 ms. In addition, the above-mentioned times are summarized in Table 1.

### 4.2. Vibration Type Identification

Subsequently, the experiments concerning vibration type identification were also conducted to verify the proposed method. A PZT cylinder with 60 cm fiber wounded was put around 1 km over a total fiber length of 1.2 km as the vibration source. In the experiment, the PZT cylinder was driven by a signal generator (DG1022U, RIGOL, Beijing, China), whose modulation frequency and amplitude can be adjusted.

#### 4.2.1. Sine Wave Signal Test

**Figure 6:**
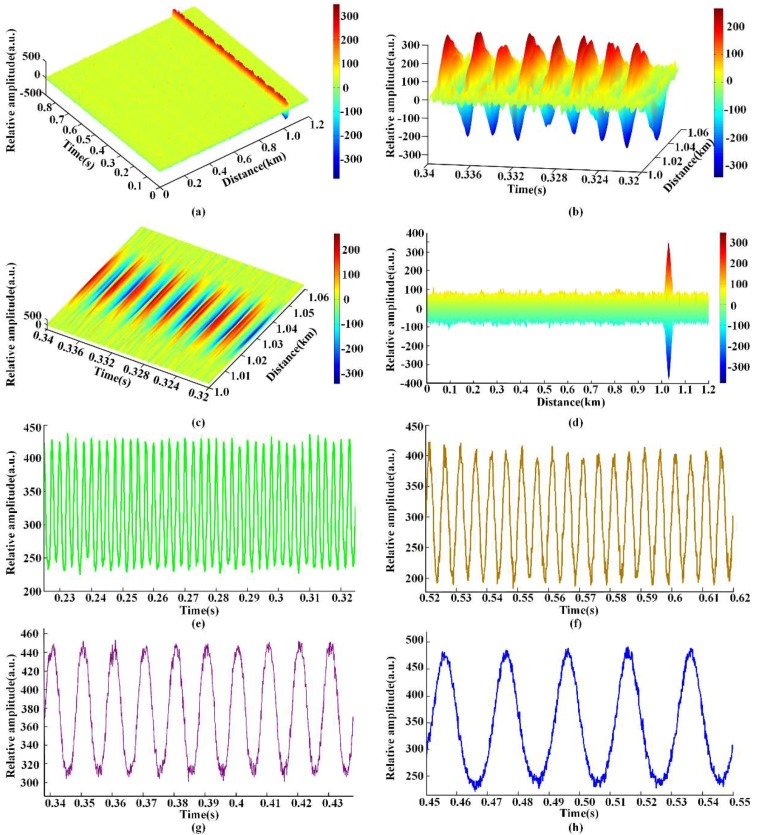
Sine wave signal test: (**a**) 3D plot of the intensity variation under 400 Hz sinusoidal signal; (**b**) partially enlarged view of the intensity variation under 400 Hz sinusoidal signal; (**c**) top view of the intensity change under 400 Hz sinusoidal signal; (**d**) superimposed differential signal under 400 Hz sinusoidal signal; (**e**) extracted vibration waveform under 400 Hz sinusoidal signal; (**f**) extracted vibration waveform under 200 Hz sinusoidal signal;(**g**) extracted vibration waveform under 100 Hz sinusoidal signal; (**h**) extracted vibration waveform under 50 Hz sinusoidal signal.

In our experiment, sinusoidal signal test was firstly carried out to evaluate the proposed method. A sinusoidal voltage of 20 v_p-p_ with a frequency of 400 Hz was applied to the PZT cylinder. Using the arrangement in Figure 4, continuous raw 7000 RBS traces were recorded during 875 ms time interval. Then, the recorded traces were processed in the software and the experimental results were shown in Figure 6a–e. Moreover, sine waves of frequencies of 200 Hz, 100 Hz and 50 Hz were also tested and the results were given in Figure 6f–h.

Figure 6a shows the 3D plot of the detected optical intensity variation versus time and distance. It can be obviously seen that strong intensity variation has been appeared around 1 km, while basically remains constant at other positions. In addition, partially enlarged view and top view (0.32~0.34 s; 1~1.06 km) are presented in Figure 6b,c. Continuous intensity variation with peaks synchronized with the applied frequency can be observed. Afterwards, the superimposed differential signal of the acquired 7000 RBS traces is shown in Figure 6d, and the detected vibration position is 1.028 km, with a SNR of 10.45 dB. Finally, the vibration waveform is extracted and shown in Figure 6e, and a clear sinusoidal signal with a frequency of 400 Hz is successfully detected. In addition, experiments of sinusoidal signals with different frequencies of 200 Hz, 100 Hz and 50 Hz were also conducted, and the experimental results are presented in Figure 6f–h. Therefore, the experimental results prove the effectiveness of the proposed method.

#### 4.2.2. Square Wave Signal Test

A square wave signal test was also carried out. In the experiment, a square voltage of 5 v_p-p_ with a frequency of 50 Hz was applied to the PZT cylinder. Similar to the sine wave test, continuous 7000 raw RBS traces were continuously recorded during 875 ms time interval. 

**Figure 7:**
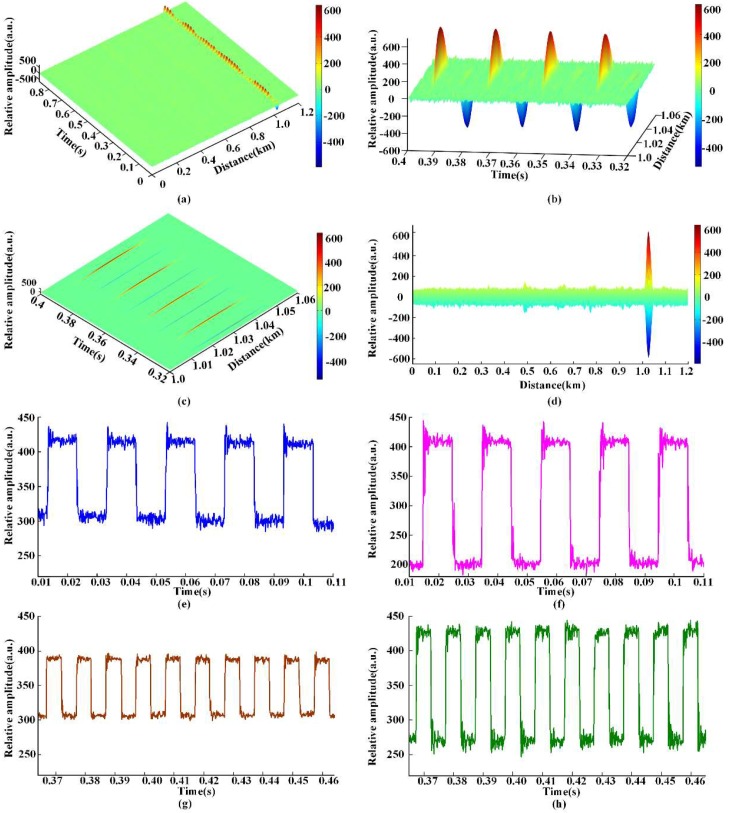
Square wave signal test: (**a**) 3D plot of the intensity variation under 50 Hz square wave signal; (**b**) partially enlarged view of the intensity variation under 50 Hz square wave signal; (**c**) top view of the intensity variation under 50 Hz square wave signal; (**d**) superimposed differential signal under 50 Hz square wave signal; (**e**) extracted vibration waveform under 50 Hz 5 V_pp_ square wave signal; (**f**) extracted vibration waveform under 50 Hz 10 V_pp_ square wave signal;(**g**) extracted vibration waveform under 100 Hz 5 V_pp_ square wave signal; (**h**) extracted vibration waveform under 100 Hz 10 V_pp_ square wave signal.

Afterwards, the recorded traces were processed in the software and the experimental results are presented in Figure 7a–e. Moreover, square waves of different frequencies and amplitudes were also tested and the results were given in Figure 7f–h. Figure 7a shows the 3D plot of the intensity variation versus time and distance. Similarly, strong intensity variation around 1 km can be observed, while basically remains constant in other positions. Partially enlarged view and top view (0.32~0.4 s; 1~1.06 km) are shown in Figure 7b,c. Impact intensity variation which is caused by the rising edge and falling edge of the square wave can be observed. Then, superimposed differential signal of the RBS traces is shown in Figure 7d, the detected vibration position is 1.026 km and the SNR is 12.62 dB. Finally, the vibration waveform is extracted and shown in Figure 7e, a clear square signal with a frequency of 50 Hz is successfully recovered. Moreover, experiments of square signals of 50 Hz 10 V_PP_, 100 Hz 5 V_PP_, and 100 Hz 10 V_PP_ were conducted and shown in Figure 7f–h. Thus, the experimental results further verifies the effectiveness of the proposed data matrix matching method.

## 5. Conclusions

In this work, an efficient data matrix matching method is proposed and successfully employed in the Φ-OTDR system to achieve real-time vibration location and type identification. On one hand, real-time vibration detection is implemented in visual C++ based on the technique of dual-thread mechanism, and the response time is improved to millisecond level for the first time. On the other hand, vibration type identification is obtained by utilizing the proposed method. Preliminary experiments are carried out and the results prove the feasibility and validity of the proposed method. Vibration signals can be detected and located in real time over a total fiber length of 1.2 km with an average response time of 50.1 ms, under a data transmission speed which can go up to 77.824 Mbps. Moreover, different vibration types such as sine waves and square waves can also be successfully identified. Therefore, this new method provides a reliable and cost-effective solution for monitoring vibration information comprehensively and hence may find applications in many practical situations.

Also, more effort is still needed to further improve the sensing performance. For the HDIF used in our system, the maximum transmission speed is approximately 300 Mbps, tested by the Cypress official program. Since the pulse repetition rate is 8 kHz, thus, the maximum fiber length under the data matrix matching method is 4.7 km. Currently, we choose 1.2 km to establish the feasibility of the concept, and we are also trying to integrate faster data transmission interface and optimized algorithms for longer distance real-time vibration location and type identification.

## Figures and Tables

**Table 1:** Summary of the times.

Parameters	Value	Description
*t*_1_	12 μs	light propagating time in fiber
*t*_2_	125 μs	pulse repetition period
*t*_3_	0.02 μs	A/D sampling time
*t*_4_	125 μs	transmission time from HDIF to host computer
*t*_5_	47.6~52.6 ms	refresh time of the MFC interface
